# Characterization of canine adipose- and endometrium-derived Mesenchymal Stem/Stromal Cells and response to lipopolysaccharide

**DOI:** 10.3389/fvets.2023.1180760

**Published:** 2023-05-19

**Authors:** Hlaing Phyo, Amira Aburza, Katie Mellanby, Cristina L. Esteves

**Affiliations:** The Roslin Institute and R(D)SVS, The University of Edinburgh, Edinburgh, United Kingdom

**Keywords:** MSC, veterinary MSC, dog, canine, regenerative, repair, differentiation, LPS

## Abstract

Mesenchymal stem/stromal cells (MSCs) are used for regenerative therapy in companion animals. Their potential was initially attributed to multipotency, but subsequent studies in rodents, humans and veterinary species evidenced that MSCs produce factors that are key mediators of immune, anti-infective and angiogenic responses, which are essential in tissue repair. MSCs preparations have been classically obtained from bone marrow and adipose tissue (AT) in live animals, what requires the use of surgical procedures. In contrast, the uterus, which is naturally exposed to external insult and infection, can be accessed nonsurgically to obtain samples, or tissues can be taken after neutering. In this study, we explored the endometrium (EM) as an alternative source of MSCs, which we compared with AT obtained from canine paired samples. Canine AT- and EM-MSCs, formed CFUs when seeded at low density, underwent tri-lineage differentiation into adipocytes, osteocytes and chondrocytes, and expressed the CD markers CD73, CD90 and CD105, at equivalent levels. The immune genes IL8, CCL2 and CCL5 were equally expressed at basal levels by both cell types. However, in the presence of the inflammatory stimulus lipopolysaccharide (LPS), expression of IL8 was higher in EM- than in AT-MSCs (*p* < 0.04) while the other genes were equally elevated in both cell types (*p* < 0.03). This contrasted with the results for CD markers, where the expression was unaltered by exposing the MSCs to LPS. Overall, the results indicate that canine EM-MSCs could serve as an alternative cell source to AT-MSCs in therapeutic applications.

## Introduction

Mesenchymal stem/stromal cells (MSCs) are multipotent cells used in regenerative therapy in companion animals. MSCs have been classically obtained from adipose tissue (AT) and bone marrow (BM) (1–7) but other tissues, including endometrium (EM) (8–10), Wharton’s jelly (11) and umbilical cord blood (12) have also been used as the source of these cell preparations. Previous studies in human and veterinary MSCs (8, 13–16), exploring different tissue sources (16, 17), showed that although MSCs share many similar properties, they also evidenced individual features depending on the tissue of origin. Indeed, when compared to AT-MSCs, equine EM-derived MSCs (EM-MSCs) have distinct immune (17) and transcriptomic signatures (14), possibly consequence of being a tissue naturally exposed to infection and inflammation.

Traditionally, veterinary MSCs have been defined following the International Society for Cellular Therapy (ISCT) guidelines for human cells, namely on the expression of cell surface markers CD73, CD90 and CD105 and ability of these cells to undergo trilineage differentiation into adipocytes, chondrocytes and osteocytes (18, 19). Although valuable, some of the initial ISCT guidelines proved difficult to apply to veterinary species, commonly consequence of the natural absence of expression of particular CD markers or due to technical difficulties associated with the lack of appropriate antibodies (20, 21). Over the years, it also became clear that these criteria did not necessary contemplate or correlate with cell function. Therefore, toward a better definition and standardization of veterinary MSC preparations, different groups published guidelines, based essentially in equine and to a less extent in canine data, to help addressing this issue (22, 23).

In addition to cell differentiation capability, MSCs produce diverse angiogenic and immune factors (24) which are relevant during repair and anti-infective body responses. Altogether, these findings stimulated great interest in the use of MSCs for different therapeutic applications in companion animals, for example on joint disease, wound infection, chronic gingivostomatitis, atopic dermatitis, multidrug resistant infections, among others (25–29).

Whilst MSCs from humans and veterinary species, particularly from horses but also from dogs (23, 30), have been extensively studied toward their therapeutic use in inflammation-associated disease, namely in cartilage degeneration and osteoarthritis, considerably less work has been done on the anti-infective properties of MSCs, which is an area that has just recently started being explored. Relevant to both inflammatory-and infection-associated settings is the communication between MSCs and immune cells. Indeed, MSCs are highly responsive to inflammatory stimuli, for example when exposed to cytokines IL1β, TNFα, IL8 and IL6 (31, 32). Likewise, infectious products such as bacterial lipopolysaccharide (LPS), polyinosinic:polycytidylic acid or the peptidoglycan dipeptide iE-DAP (17, 31, 33) activate Toll-like and nucleotide-binding oligomerization domain (NOD)-like receptors (TLR, NLR), respectively, resulting in increased expression of immune modulators and antimicrobial factors (17, 31, 34, 35). Indeed, activation of human and equine MSCs with LPS, upregulates the expression of chemokines such as CCL2, CCL5, IL8 and IL6 (17, 34, 35), which are involved in recruitment and maturation of immune cells, neutrophils and monocytes (36–38). LPS is a toxin present in the outer membrane of Gram-negative bacteria such as *Escherichia coli*, which is frequently associated with canine infection, for example of the urinary tract. Testing canine MSC response to LPS will inform on the behavior of these cells in an infectious context, namely on their response upon LPS activation *in vitro*. Importantly, priming of MSCs with LPS was shown to be of benefit both *ex vivo*, and in *in vivo* studies involving rodent models of disease (39, 40), therefore supporting activation of MSCs as a way to enhance the properties of these cell preparations.

Considering what was described above, in this study we compared canine MSCs derived from two tissue sources, EM and AT. In addition to the standard MSC characterization, and to compare EM- and AT-MSCs further, we measured a selected group of immune factors (IL8, CCL2 and CCL5) that were expressed in these cells at basal levels and, as we have previously assessed in equine MSCs, were induced by LPS.

## Materials and methods

### Extraction of canine MSCs from endometrium and adipose tissue

Samples were obtained from spare tissues of the reproductive tract of female dogs (*n* = 3; Supplementary Table S1) undergoing sterilization at the Royal (Dick) School of Veterinary Studies, following approval by the Ethical Review Committee, University of Edinburgh. For each animal, ovaries, uterine horns and uterine body were removed as one piece and immediately transported (on ice) to the laboratory to be processed. A solution of cold phosphate buffered saline (PBS) with 1% of penicillin/streptomycin mix (P/S; Life Technologies), and 5 μg/ml amphotericin B (Gibco-Thermo Fisher Scientific) was used for washing the tissues. Then, the uterine body and horns were cut longitudinally with a scalpel to obtain the EM by scraping the tissue, and the AT surrounding the reproductive tract was also harvested for extraction, from each animal. The collected tissues were washed, minced and digested. EM was digested with collagenase I (5 mg/ml; Gibco-Thermo Fisher Scientific, 17100-017) and AT with collagenase II (1 mg/ml, Gibco-Thermo Fisher Scientific, 17101-015) for 45 min at 37°C, and under constant moderate agitation (70 rpm). Collagenase activity was stopped with Dulbecco’s Modified Eagle’s Medium (DMEM) high glucose (Sigma-Aldrich) supplemented with 20% fetal bovine serum (FBS) (Gibco-Thermo Fisher Scientific). Cells were then filtered through a 100 μm strainer and cultured in DMEM containing 20% FBS, 1% of P/S at 37°C. Pictures were taken using a Nikon Eclipse TE2000U Microscope. All experiments were performed with MSCs grown between passages 3–5.

### Clonogenicity

To obtain colony forming units (CFUs), 500 cells/well were seeded in 6-well plates. Cells were grown for 10 days in complete growth medium DMEM (Sigma-Aldrich) containing 20% FBS (Gibco-Thermo Fisher Scientific) and 1% of P/S (Life Technologies). After that, CFUs were washed with PBS, fixed with PFA (2%; 30 min) and stained with 0.5% crystal violet for 30 min.

### Cell differentiation

For adipogenic differentiation (41), MSCs were seeded in triplicate in 24-well plates (50,000 cells/well) and expanded in growth medium until confluence. Adipogenesis was induced using the medium containing 10% FBS, 1 μM dexamethasone (Sigma-Aldrich), 0.5 mM isobutylmethylxanthine (Sigma-Aldrich), 10 μg/ml insulin (Sigma-Aldrich), 100 μM indomethacin (Sigma-Aldrich), 1% P/S in DMEM. Cells were kept in differentiation medium for 6 days and then changed to 10% FBS, 10 μg/ml insulin and 1% P/S for a total of 14 days. Cell growth medium was used for the control cell group. Both differentiated and control MSCs were fixed with 4% paraformaldehyde before visualization of lipid droplets by Oil Red O staining. Imaging was performed in a Zeiss Axiovert 25 Inverted Phase microscope using Zen 2 software (Advanced Micro Devices).

Osteogenesis was induced with a mixture of DMEM high glucose and DMEM low glucose (50:50 v/v; Sigma-Aldrich), supplemented with 10% FBS, 100 nM dexamethasone (Sigma-Aldrich), 10 mM sodium β-glycerophosphate (Sigma-Aldrich) and 0.1 mM stabilized ascorbic acid (Sigma-Aldrich). After 3 days, cells were changed to DMEM low glucose medium with the same supplements. Cells were cultured for 19 days and medium was changed every 3 days. At the end of the differentiation period cells were fixed with 4% paraformaldehyde, stained with Alizarin Red (2%; pH 4.2) and imaged in a Zeiss Axiovert 25 Inverted Phase microscope using Zen 2 software (Advanced Micro Devices).

For chondrogenesis, MSCs were suspended in a small volume of media to generate a concentrated cell solution of 1.6 × 10^7^ cells/ml. Micromass cultures, 5 μl droplets of this cell suspension, were seeded in 96-well plates and incubated at 37°C for 2 h under high humidity conditions. STEMPRO^®^ chondrogenesis differentiation media (Gibco, Fisher Scientific) was then added to the micromasses and refreshed every 2–3 days. Cell growth media was used for controls. After 16 days, cells were fixed with 4% formaldehyde for 30 min and stained with 1% alcian blue solution prepared in 0.1 N HCL.

### LPS stimulation experiments

MSCs were plated in 12-well plates (75,000 cells/well) and kept for 48 h, prior to incubation for 16 h with 0.1 μg/ml lipopolysaccharide from *Escherichia coli* O111:B4 (Sigma, L2630), alongside with unstimulated control cells. AT- and EM-MSCs were harvested with Trizol and immediately stored at −80°C prior to RNA extraction.

### RNA extraction and cDNA synthesis

RNA was extracted from cells in Trizol following manufacture’s protocol and reverse transcribed into cDNA using Superscript III (Invitrogen-Thermo Fisher Scientific). A NanoDrop 1000 Spectrophotometer (Thermo Scientific, Wilmington, United States) was used to measure the quality and concentration of RNA. Negative controls were produced either without RNA sample or superscript enzyme.

### Quantitative PCR analysis

Gene transcript levels were quantified by qPCR using the primers listed in Table 1 using SensiFAST SYBR Lo-ROX kit (Bioline) in a MX3005P thermocycler (Stratagene), using the conditions, Step 1: denaturation at 95°C for 2 min, followed by Step 2: 40 cycles of, 95°C for 5 s, 60°C for 11 s, 72°C for 5 min. Step 3: Final extension; 95°C for 1 min, 60°C for 30 s, 95°C for 30 s. Results were analyzed with MxPro software (Stratagene) relative to a standard curve obtained from a pool of cDNA samples. Two housekeeping genes (18S and GAPDH) were used to normalize the individual gene expression results.

**Table 1:** Primers used in qPCR.

Gene	Sequence (5′ to 3′)
CD45	F: TCGGCTTTGCCTTTCTGGAT
	R: TTCTGGGGAAACAGAACTGGA
CD73	F: TACACAGGTACTCCACCTTCCA
	R: AACCTTCCGCCCATCATCAG
CD90	F: AGGACGAGGGGACATACACA
	R: CTTGACCAGTTTGTCTCTGAGC
CD105	F: CCTGGAATCCTCAAGGGAGC
	R: ACTGAGGACCAGGAACACCT
IL8	F: TGTGAAGCTGCAGTTCTGTCAA
	R: TTGGGATGGAAAGGTGTGGAG
CCL2	F: AAGCTGTGATCTTCAAGACCGT
	R: CATGGAATCCTGGACCCACT
CCL5	F: CAGTCGTCTTTGTCACCCGA
	R: TGTACTCCCGCACCCATTTC
18S	F: GCTGGCACCAGACTTG
	R: GGGGAATCAGGGTTCG
GAPDH	F: GCCTGGAGAAAGCTGCCAAA
	R: TTTGAGGGGTCCCTCCGATG

F and R stands for forward and reverse primers, respectively.

### Statistical analysis

Results were analyzed by Student’s *t*-test, or two-way ANOVA followed by LSD *post hoc* test as appropriate, by using GraphPad Prism 9. Statistical significance was set at *p* < 0.05.

## Results

### Characterization of AT- and EM-derived MSCs

**Figure 1:**
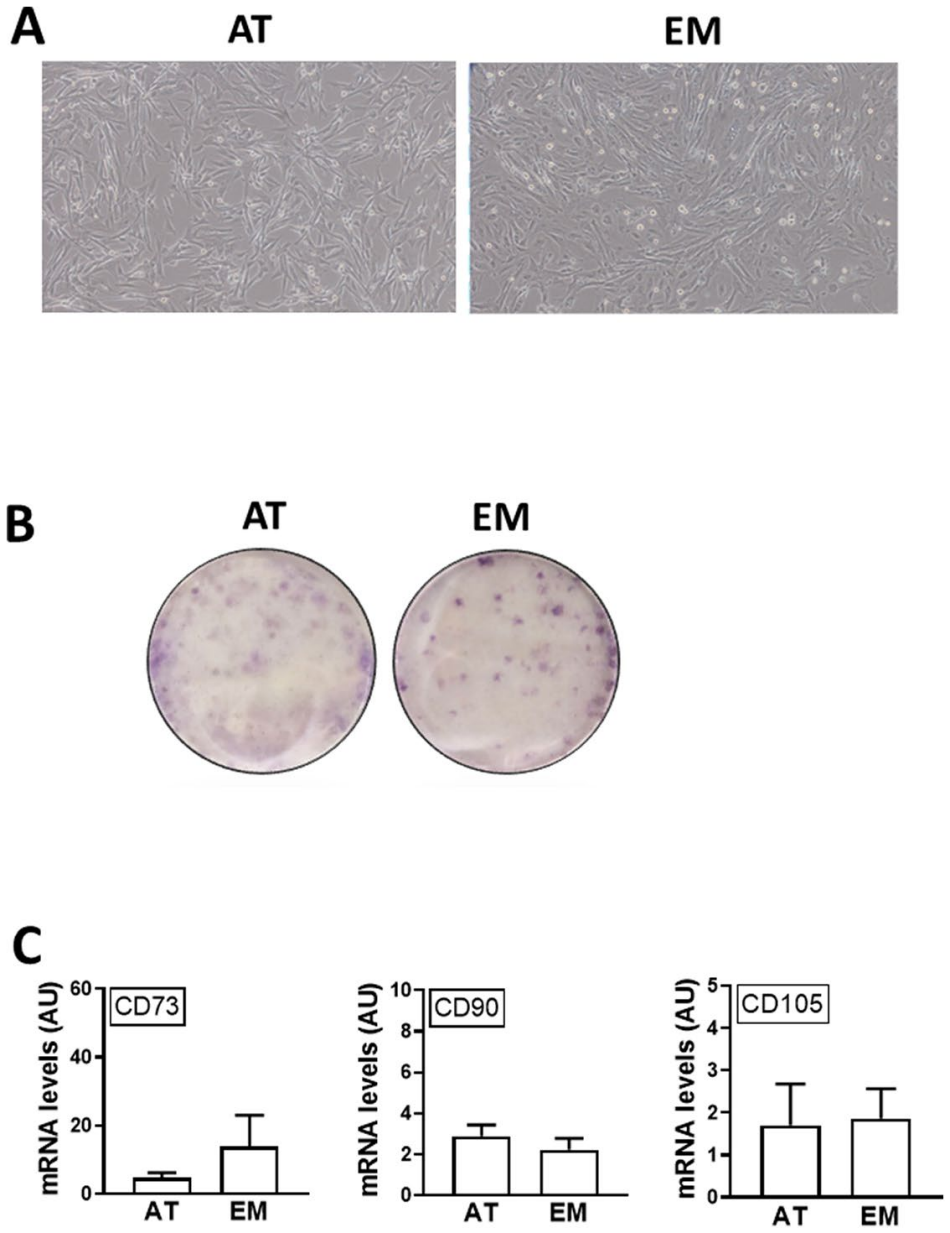
Characterization of AT- and EM-derived MSCs. **(A)** Micrographs of cells in culture taken at 10x magnification. **(B)** Cell colonies obtained from 500 cells/well and stained with crystal violet after 10 days in culture. **(C)** Expression levels of CD markers CD73, CD90 and CD105 quantified by qPCR. All results are shown as mean ± SEM; AU, arbitrary units.

**Figure 2:**
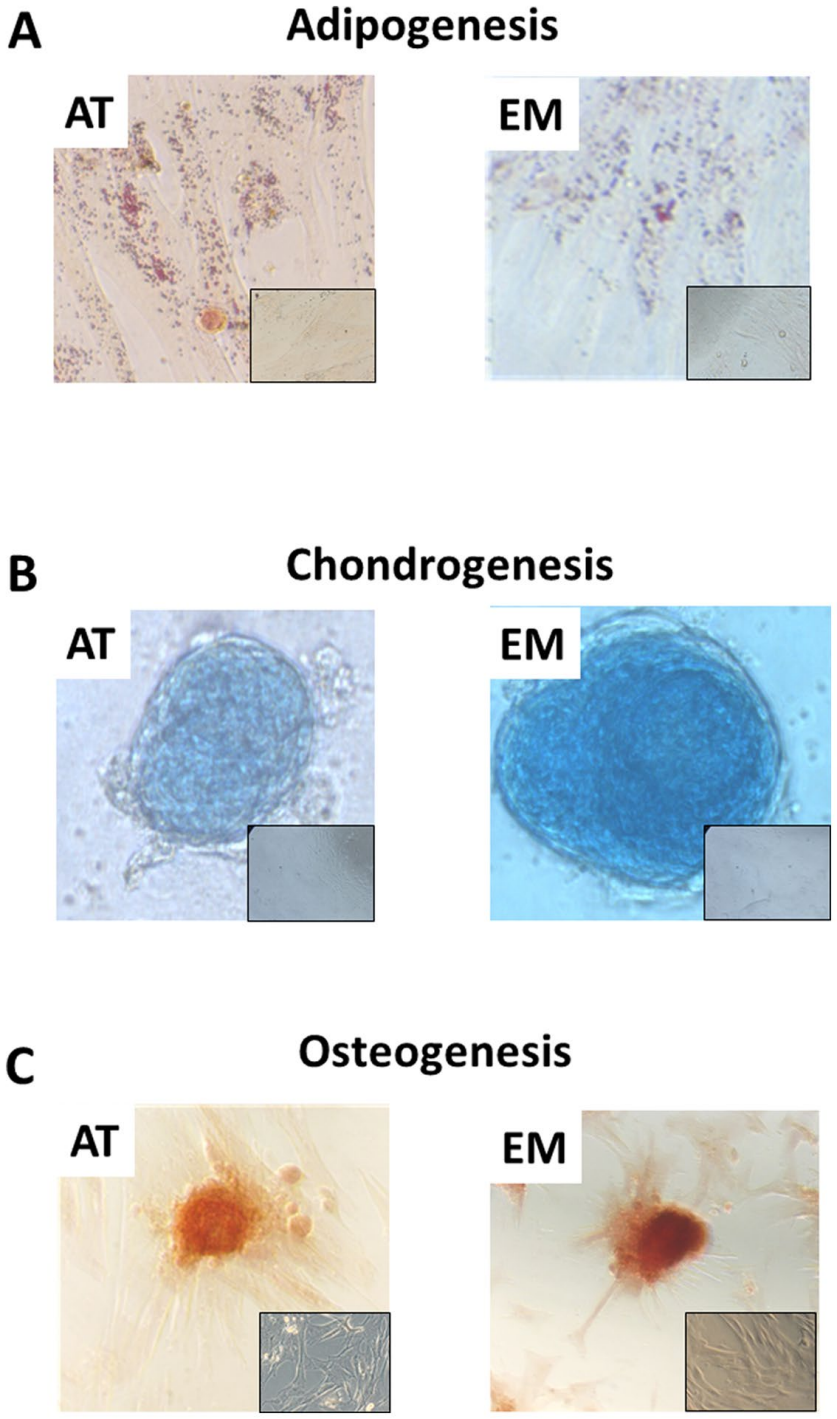
Tri-lineage differentiation of AT- and EM-MSCs. Micrographs showing **(A)** adipocytes, **(B)** chondrocytes and **(C)** osteocytes stained with Oil red O, Alcian blue and Alazarin Red, respectively. Insets show control non-differentiated cells. Micrographs were taken at 40× magnification.

Both AT- and EM-MSCs growing in culture presented the typical spindle-like morphology of MSCs (Figure 1A), form CFUs (Figure 1B) and expressed CD markers CD73, CD90 and CD105 (Figure 1C) at similar levels, while CD45 was undetectable in both cell types. After incubation with adipogenic media, AT- and EM-MSCs gradually changed their morphology and lipid droplets accumulated as shown by oil red O staining (Figure 2A). In MSCs undergoing osteogenesis alizarin red staining evidenced the deposition of calcium in the differentiated cells (Figure 2C). For chondrogenesis, MSCs cultured at high cell density as micromasses acquired a round morphology, and differentiation was confirmed by alcian blue staining 16 days following the start of differentiation (Figure 2B). No differences were observed between EM- and AT-MSCs differentiated cells.

### Gene expression of MSC markers was unchanged by stimulation of AT- and EM-MSCs with LPS

**Figure 3:**
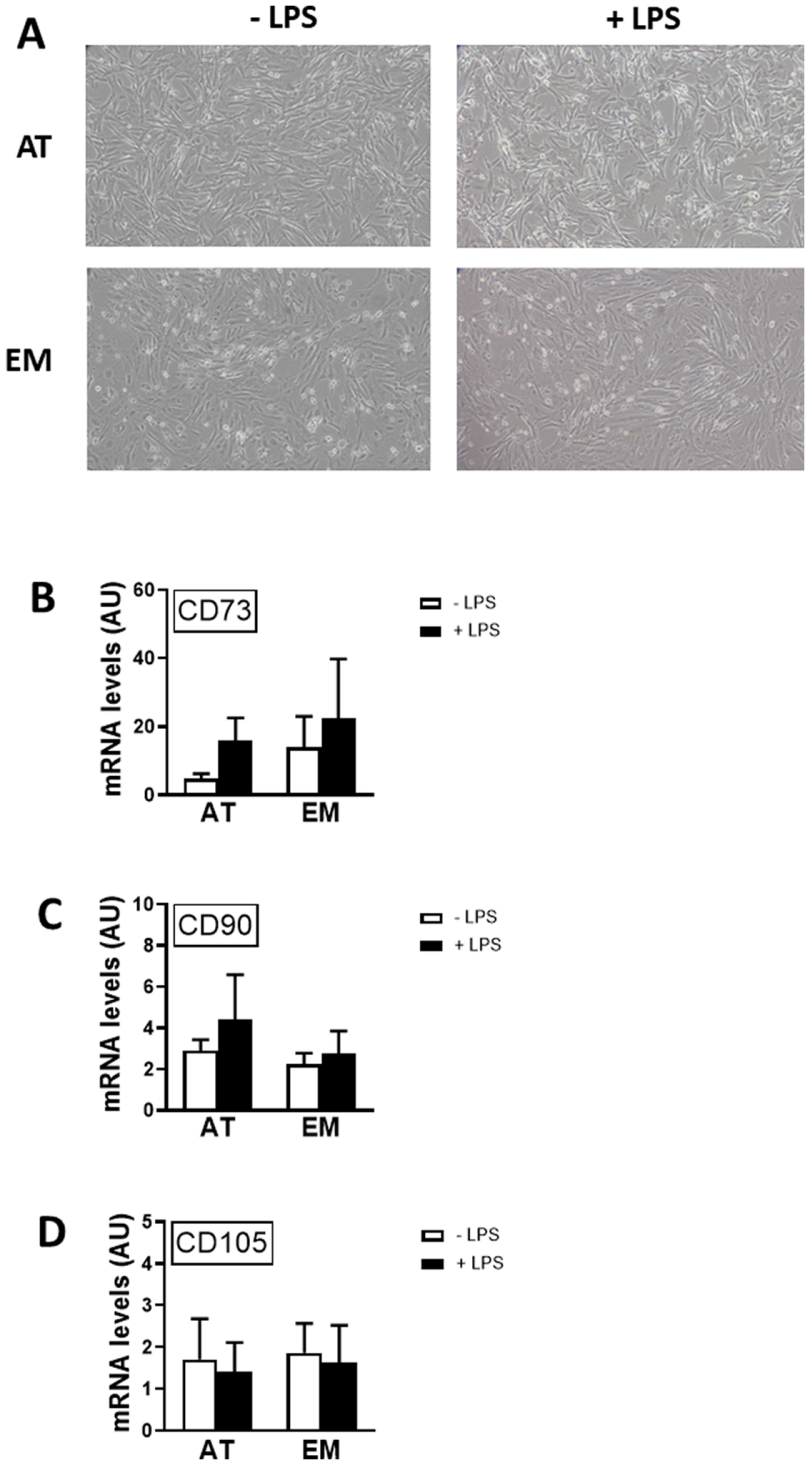
Effect of LPS on the expression of MSC markers in AT- and EM-MSCs. **(A)** Micrographs of control cells (− LPS) or stimulated with LPS (+ LPS). **(B–D)** Expression levels of the CD markers CD73, CD90 and CD105 quantified by qPCR, of controls (− LPS; white bars) versus LPS-stimulated cells (+ LPS; black bars). All results are shown as mean ± SEM; AU, arbitrary units.

In order to test if MSC markers were affected by cell activation, gene expression of CDs 73, 90 and 105 was measured by qPCR following incubation of cells with LPS for 16 h. LPS treatment did not affect the morphology of the cells, as shown in Figure 3A, and did not cause variation in gene expression levels of CDs 73, 90, and 105 in both AT- and EM-MSCs (Figures 3B–D). Likewise, no differences were observed between AT- and EM-MSCs CDs expression levels (Figures 3B–D).

### Gene expression of immune mediators in MSCs was increased by LPS

**Figure 4:**
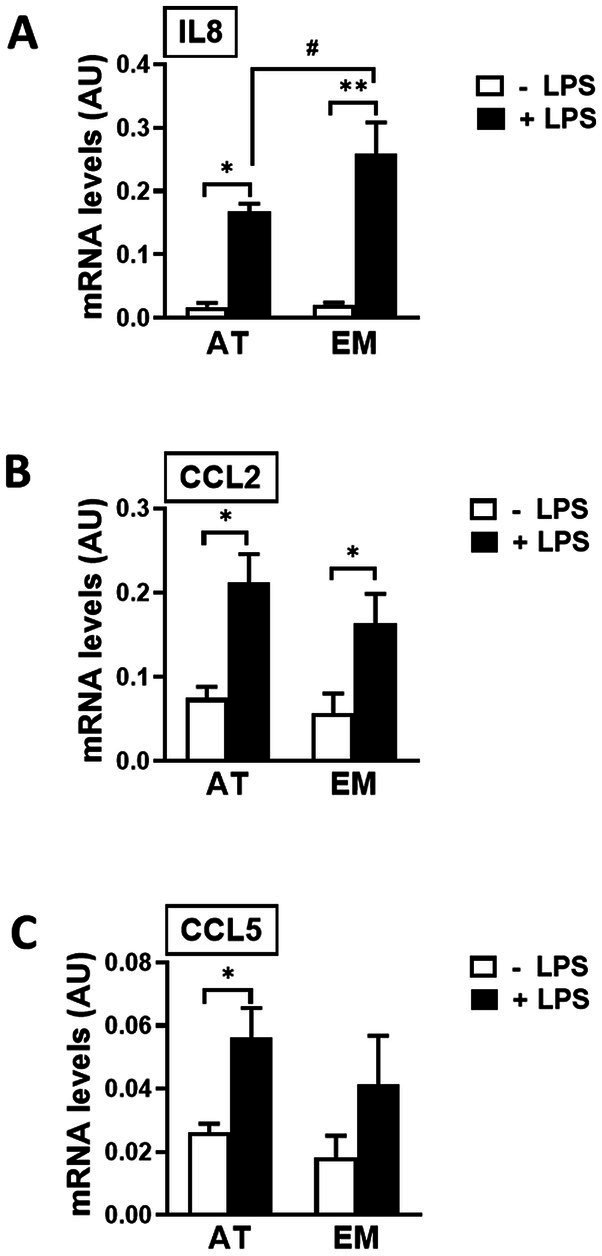
Expression of immune mediator genes in AT- and EM-MSCs activated with LPS. mRNA levels of **(A)** IL8, **(B)** CCL2 and **(C)** CCL5 measured by qPCR of control (− LPS; white bars) and LPS-stimulated MSCs (+ LPS; black bars). All results are shown as mean ± SEM; AU, arbitrary units. * and **p < 0.05 and < 0.001, respectively, of control versus LPS-treated cells. ^#^*p* < 0.05 of AT- versus EM-MSCs.

Both cell types, AT- and EM-MSCs, expressed the cytokine IL8 and chemoattractants CCL2 and CCL5 at similar basal levels (Figures 4A–C). Those genes were significantly increased when cells were exposed to LPS (*p* < 0.03), except for CCL5 in EM-MSCs where gene expression was not significantly altered. In the presence of LPS, IL8 values were higher in EM-than AT-MSCs (*p* < 0.04), while no other differences were observed between the MSCs obtained from these two tissue sources.

## Discussion

In this study, we compared MSCs from two different tissue origins, EM and AT, obtained from the reproductive tract of healthy female dogs undergoing sterilization. EM- and AT-MSCs in culture displayed the standard spindle-like shape, formed CFUs when seeded at low cell density, expressed CD markers (CD73, CD90 and CD105) and underwent tri-lineage differentiation at similar level. Of note, both EM- and AT-MSCs expressed the cytokines IL8, CCL2 and CCL5 at basal levels. Therefore, to further characterize and compare the canine MSCs, and based on previous results in equine MSCs showing differential expression of these cytokines (17), we measured the effect of LPS on the expression of IL8, CCL2, and CCL5, in EM- and AT-MSCs. This resulted in elevated values of IL8, CCL2 and CCL5 in LPS-induced MSCs, with levels of IL8 for EM-MSCs being significantly higher than for AT-MSCs. Contrary to these results, CD marker levels remained unchanged upon priming of the cells with LPS, and therefore CD marker expression did not correlate or reflect cell activation.

Canine AT- and EM-MSCs displayed comparable cell features as defined by the ISCT, in agreement to what was observed in equine and human MSCs (13, 17). Indeed, MSCs obtained from different tissue origins share a variety of common features (42), including expression of CD markers and tri-lineage differentiation (8). Expression of CD markers in MSCs is sustained in different conditions, for example when human BM-MSCs are maintained in culture for an extended period of time, although other features including cell morphology, doubling time and osteogenic differentiation are affected (43). Likewise, culturing of equine BM-MSCs in platelet lysate improved chondrogenesis compared to FBS, but did not change the MSC markers CD105 and CD44 (44), and canine BM-MSCs cultured in the presence of FBS had significant higher survival rate compared to cells cultured in serum-free conditions, while CD marker expression levels were unaffected (45). Similarly, in the present study we observed that both AT- and EM-MSCs expressed CD73, 90 and 105 at equivalent basal levels, which were unchanged in the presence of LPS, but immune mediator genes were upregulated upon MSC stimulation with LPS. These results support the idea that expression of CD markers cannot be used as reliable indicators of stem cell content or biological function of MSC preparations. Different surface markers, including Stro-1, SSEA-4, CD271, and the pericyte marker CD146 have been considered as candidates (46) for this purpose. CD146 is present both in MSCs and pericytes, including in the horse (1, 7, 47) where it has been used to isolate cells with superior angiogenic potential compared to the corresponding MSC preparations. Still, considerable more work needs to be done, especially in veterinary species, toward the establishment of proper guidelines for a better characterization and standardization of MSCs preparations, although attempts in this direction have already been made by different groups, especially for equine MSCs (20, 22, 23).

A diverse number of studies in humans, rodents and veterinary species (principally in horses but also in dogs) have tested the effect of priming MSCs with inflammatory and infectious stimuli (24, 48, 49) in order to assess MSC response to disease milieu and to enhance their therapeutic properties (50). Human and rodent MSCs (principally obtained from BM and AT, but also from other sources) primed with a variety of activators such as TNFα, IL1β, IFNγ, IL17A, LPS and Poly I:C showed increased MSC expression of immune modulators, antibacterial peptides, growth and angiogenic factors. TNFα, alone or combined with IL1β, increases the levels of IL6, VEGF, FGF2, IGF-1, and HGF (51, 52), while IFNγ was shown to increase CCL2, IDO, TGFβ, HGF (53, 54), and IL17A to elevate IL6 (55). Infectious stimuli, such as LPS increases pro-inflammatory molecules including CXCL1, IL8, IL6, CCL2 and LL37 (56) while activation of TLR3 results in upregulation of IDO and PGE-2 (57). These findings show the diversity of phenotypes that can be generated by MSC activation depending on the stimulus used.

It is evident from the literature that a diverse number of studies tested inflammatory stimuli with just a few assessing infectious- or bacterial-associated activation. This, together with our previous results in different equine MSCs types, prompted us to follow the same approach here, as the main objective of this study was to compare canine EM- and AT-MSCs properties, although the use of LPS as a single stimulant is a limitation in this study. Indeed, since MSCs are highly responsive to a variety of stimuli, not only immune- and infectious-related but also a diversity of others, additional inducers could be included in future work in order to cover a broader range of responses comparing canine EM- and AT-MSCs. In addition, aiming for a wider characterization, comparison between canine EM- and AT-MSCs could be further complemented by comprehensive gene expression and protein analysis of MSC responses by performing RNA sequencing, LC–MS-based proteomics, or multiplex immunoassays, and we are planning to perform these experiments in future studies.

Similarly to humans and rodents, preconditioning of equine BM-MSCs with IL1β resulted in increased expression of IL1β, IL6, IL8, but not of IL10 and TNFα, while the combine action of TNFα and IFNγ resulted in an anti-inflammatory phenotype, with increased expression of COX-2, iNOS, IDO, IL6 (32), which effect has also been observed in human MSCs (58). CCL2 was also elevated by TLR3 activation when equine BM-MSC were stimulated with poly I:C (33), but priming with LPS increases CCL2, IL8 and IL6, showing that equine MSCs are highly responsive to bacterial wall components, part of the indirect but relevant role of these cells in modulating the immune response to combat infection.

Likewise, expression of immune genes is altered when canine MSCs are activated, for example COX-2 increases when AT- and BM-MSCs are stimulated with TNFα and IFNγ (59), and priming of AT-MSCs with deferoxamine, a hypoxia-mimetic agent, potentiates anti-inflammatory effects in RAW 264.7 macrophages (60). Also, stimulation of canine MSCs with TNFα elevates TSG-6 and PGE2 resulting in *in vivo* benefit by regulating colonic inflammatory cytokines such as IL1β, IL6, and IL10, and ameliorating induced colitis in mice (61). However, compared to other species, work performed on canine MSC activation is limited.

Here we showed that stimulation of canine MSCs with LPS increased the expression of immune genes IL8, CCL2 and CCL5, except CCL5 in EM-MSCs that was not upregulated. Of note was that in both equine (17) and canine EM-MSCs the expression of CCL2, but not CCL5, was significantly induced by LPS. This indicates that these chemoattractants are differentially induced by LPS in EM-MSCs, at least in these veterinary species. Also, EM-MSCs expressed IL8 at higher levels compared to AT-MSCs, following cell stimulation with LPS. Indeed, the immune properties of MSCs may vary depending on the tissue of origin (8, 42, 62, 63). Equine EM-MSCs activated with LPS express IL6 at higher levels than AT-MSCs (17) and human dental MSCs express INF-γ, PDGFA, VEGF and IL10 more elevated levels than BM-MSC (64), while human AT-MSCs produce higher levels of IL6 and TGF-β1 than BM-MSCs. In contrast, IFNγ treatment of bovine BM- and AT-MSCs increased IL6, PTGER2 and IDO gene expression at similar levels (65), indicating that species and type of stimulus play a role in the expression of immune factors in MSCs from different tissue origins.

In this study, comparison of EM- and AT-MSCs showed that their MSC properties were similar. This agrees with previous studies in equine MSCs and suggests that EM-MSCs could serve as a viable alternative to AT-MSCs, especially given the availability of tissues resulting from routine spays. Expression of CD markers was similar in both cell types, including upon cell incubation with LPS, while the expression of cytokines was upregulated. These findings corroborate with the general position in the field that CDs are not reliable markers defining stem cell content and biological function of MSC preparations. The results also showed that both canine EM- and AT-MSCs are highly responsive to LPS demonstrated by the upregulation of cytokine gene expression, similarly to what has been previously observed in other veterinary studies, mostly in the horse. However, compared to other species, work performed on canine MSC activation is scarce. Higher levels of IL8 expression were observed in LPS-activated EM-MSCs compared to AT-MSCs, but not for other cytokines. We have previously found differential expression of immune genes in EM-MSCs compared to AT-MSCs preparations. However, how these two cell types would perform in *in vivo* repairing settings is currently unknown and warrants further studies.

## Data availability statement

The raw data supporting the conclusions of this article will be made available by the authors, without undue reservation.

## Author contributions

HP and AA performed the experiments, analyzed the results and wrote the manuscript. KM provided the samples for analysis. CE designed the study, analyzed the results and wrote the manuscript. All authors contributed to the article and approved the submitted version.

## Funding

This study was supported by a Canine Welfare Grant from Dogs Trust and the BBSRC (BB/P013732/1).

## Conflict of interest

The authors declare that the research was conducted in the absence of any commercial or financial relationships that could be construed as a potential conflict of interest.

## Publisher’s note

All claims expressed in this article are solely those of the authors and do not necessarily represent those of their affiliated organizations, or those of the publisher, the editors and the reviewers. Any product that may be evaluated in this article, or claim that may be made by its manufacturer, is not guaranteed or endorsed by the publisher.

## Supplementary material

The Supplementary material for this article can be found online at: https://www.frontiersin.org/articles/10.3389/fvets.2023.1180760/full#supplementary-material
