# Supplementary material for: Characterization of canine adipose- and endometrium-derived Mesenchymal Stem/Stromal Cells and response to lipopolysaccharide
Source: Front Vet Sci. 2023 May 19;10:1180760. doi: 10.3389/fvets.2023.1180760 (PMC10237321; doi:10.3389/fvets.2023.1180760)
Supplement: Supplementary file 1 [file Table_1.pdf]

Supplementary Table 1. Animal body parameters.

Dog	Age (years)	Weight (Kg)	Adipose Tissue (g)*
1	4	18	8.5
2	2	16	11.8
3	3	13.6	10.2

* Amount of adipose tissue obtained from each animal. Due to the collection method used to obtain endometrium it was not possible to weigh the tissue.
